# Increasing Youth Peer Workers' Impact Through Integration: Peer Worker Perspectives on Best Practice in Youth Mental Health

**DOI:** 10.1111/hex.70223

**Published:** 2025-03-17

**Authors:** Rose Singh, Sarah Leung, Vilas Sawrikar, Catherine McHugh, Nan Hu, Oliver Ardill‐Young, Raghu Lingam, Valsamma Eapen, Michael Hodgins, Jackie Curtis

**Affiliations:** ^1^ Mindgardens Neuroscience Network Sydney Australia; ^2^ Population Child Health Research University of New South Wales Sydney Australia; ^3^ Discipline of Psychiatry and Mental Health University of New South Wales Sydney Australia

**Keywords:** best practice, implementation, peer workers, young people, youth mental health services, youth peer workers

## Abstract

**Background:**

Embedding youth peer workers within youth mental health services is critical to integrating the personal lived experience of recovery‐oriented and trauma‐informed approaches in care and helping young people to make sense of the system. However, research into the optimal engagement of youth peer workers is lacking, which limits opportunities for integrating personal lived experience within care. This paper evaluated peer workers' experiences of integration into youth mental health services to identify strategies for optimal integration.

**Methods:**

Best‐fit framework synthesis was used to investigate peer worker experiences within youth mental health services using data collected from 12 peer workers through semi‐structured interviews. Themes were coded according to the key components for integrating youth peer workers into services as presented by previous guidelines (role clarity, training, supervision, professional development, agency and co‐design, non‐peer education and relationships, communication, well‐being, ongoing evaluation, remuneration and policy).

**Findings:**

Key areas of successful implementation of youth peer workers included communication networks between peers, supervision and non‐peer relationships. Areas for further development included improved role clarity, training, policy and remuneration and improvement in non‐peer staff understanding of peer roles.

**Conclusions:**

The best practice framework has utility as a model for guiding implementation evaluation of youth peer workforce into mental health services. The findings highlight the need for supporting employment pathways for youth peer workers. Further, there is a need for professional development opportunities to increase the integration of peer workers into youth mental health services.

**Patient or Public Contribution:**

One of the first authors is a youth peer worker who was involved in the design and conduct of the study, interpretation of the data and preparation of the manuscript.

More than two‐fifths (42.9%) of people aged 16–85 years have experienced a mental disorder at some time in their life in Australia, with the highest proportion of major mental and substance use disorders first emerging when adolescents transition to adulthood [1]. Worldwide, mental disorders account for 45% of years lived with disability in people aged 10–24 years, and 75% of depressive, bipolar and psychotic disorders emerge by the age of 25 [2]. To address this, there has been a rise in youth mental health services and programmes tailored to the 12–25‐year‐old age group [3, 4]. However, consumers have voiced that their needs are not being fully met, including a desire for greater flexibility in care options [5, 6]. There is a need for a systems shift to a more recovery‐oriented, patient‐centred approach to treatment that empowers young people to be involved in their treatment decisions [7, 8].

The integration of peer workers into youth mental health care is a potential solution to integrating the personal lived experience of recovery‐oriented and trauma‐informed approaches to care and helping young people make sense of the system [9, 10]. Peer workers are individuals with lived experiences comparable to those they are helping, who provide socio‐emotional support with functional goals of instilling social, vocational and personal change [11, 12]. As distinct from other kinds of mental health support, peer work is characterised by a relationship based on connection through shared experiences (‘peerness’), a reciprocal learning relationship between the peer worker and consumer, and the use of expertise through experience in the peer worker role, rather than formal or taught knowledge [13, 14, 15, 16]. The range of support offered by peer workers is diverse, including recovery planning and goal setting, help with navigating the mental health system, and individual and systemic advocacy [17, 18, 19]. Simmons et al. [20] provide a snapshot of real‐world models, which encompass individual/group, in‐person/digital and general/specific (e.g., vocational) modalities and foci. Peer support or consumer‐led services have significantly grown and have been recognised as a positive addition to mental health services for both consumers and the system at large in high‐income countries, with an emerging evidence base of implementation in low‐ and middle‐income countries [8, 21, 22, 23, 24, 25, 26, 27]. Jiang and Ramesh [28] highlights that considering the local context, including the unique goals, attitudes, formal structures and values held by the local healthcare system is key to effective implementation. In this study, we aim to understand optimal strategies for integrating peer workers in youth mental health care services in a high‐resource setting in Sydney, Australia.

Findings from qualitative research show there are unique aspects to peer workers working exclusively with young people, also known as youth peer workers (YPWs) compared to adult peer workers, given adolescence and emerging adulthood stages are times of significant change where young people are forming their identities, seeking independence and are under significant pressure socially and in the education system [11, 29]. Recent research has found that YPWs are highly promising and valued in various youth mental health treatment settings [8, 30]. YPWs can promote a diverse and inclusive workplace, reducing discrimination and stigma about mental health. By sharing their story and through advocacy for consumers' preferences and perspectives, YPWs help non‐peer staff deepen their understanding of the consumers they treat [16]. YPWs also provide emotional support to young people, building trust, validating feelings associated with struggles and adverse experiences, providing hope and advocating for them on their mental health journey.

Whilst YPWs can be valuable to the delivery of youth mental health care, implementing a specialised YPW workforce into youth mental health services has several notable challenges, and there is a lack of literature evaluating the implementation process [9, 31]. A systematic review by de Beer et al. [8] identified the key barriers and facilitators to embedding YPWs within services and found that a lack of role clarity, supervision of YPWs and peer worker well‐being were associated with difficulties in implementing peer worker programmes. As a result of these difficulties, and in part additionally because of clinician's lack of understanding of YPW or negative attitudes around integration, YPWs can be subject to negative experiences in their role [15, 32]. Addressing these core structural and cultural components is, therefore, key to supporting effective implementation and integration. However, de Beer et al. [8] noted that there was a lack of best practice principles to address these key challenges.

In an Australian local context, some organisations have developed their own framework to guide services. This includes the Orygen youth peer workforce toolkit written for the Victorian youth mental health context [33, 34]. The toolkit was developed by researchers, reflecting on evidence from the current literature on youth peer work. It lists practical tips such as developing a clear YPW role description, establishing a communication plan, delivering training and supervision, providing future career pathways, creating strong support systems and ensuring there is ongoing evaluation of the implementation. Similarly, the New South Wales (NSW) Mental Health Commission developed a guide for peer workers in the adult context [35]. They identified the importance of including peer workers in quality improvement, evaluation and design of services. They also emphasised the need for peer workers to be in policy and planning roles, management positions and system leader roles. This guide was developed by a mental health and social policy consulting agency that reviewed past literature and consulted adult peer workers. A synthesis of these recommendations across these Australian‐based guidelines into key components of implementing peer workers in mental health services is summarised in Figure 1, with component definitions provided in Table 1. For both frameworks, peer workers were consulted in the process of developing the toolkits; however, their perspectives were not systematically evaluated.

**Figure 1:**
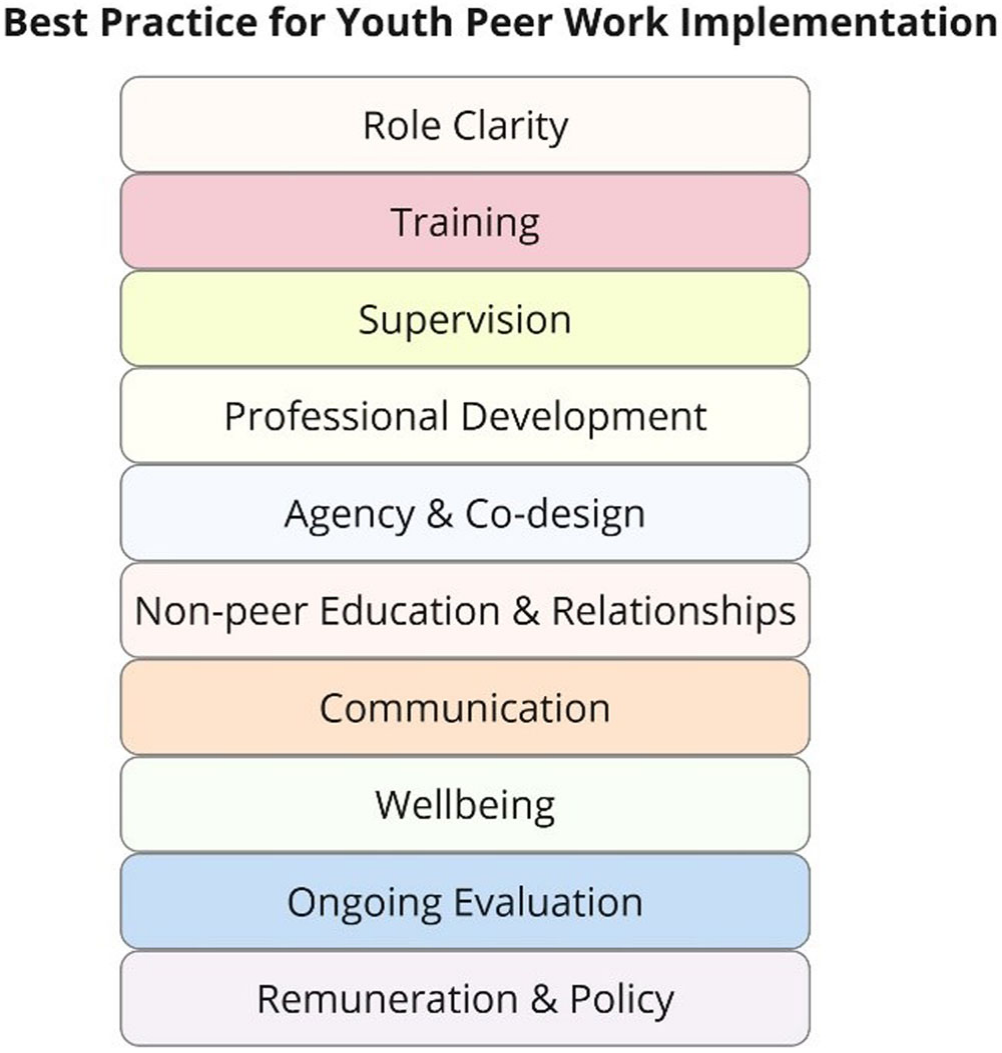
**Components of best practice for implementing a youth peer workforce**.

**Table 1:** Definitions of best practice components for youth peer work implementation.

Component	Definition
1. Role clarity	*YPWs and clinical staff having a clear understanding of the responsibilities, tasks and expectations of the YPW role.
2. Training	YPWs having formal training opportunities and resources to learn and develop skills required to perform the YPW role.
3. Supervision	YPWs having formal opportunities to seek regular support, guidance and feedback from senior trained professional/s in YPW.
4. Professional development	YPWs having formal opportunities to build more skills and abilities that will empower and enable them to progress in their future career.
5. Agency and co‐design	YPWs having opportunities to voice their opinion and be involved with designing the YPW role, training materials, evaluation, policy and more.
6. Non‐peer education and relationships	Non‐peer staff having formal education about the YPW role and how it fits into the clinical service. YPWs feeling supported and comfortable with non‐peer staff, where open discussions, team building and co‐learning can occur.
7. Communication	YPWs having resources and opportunities to communicate openly with non‐peer staff about their work and their experience in the role.
8. Well‐being	YPWs' state of mind and mental health whilst working in the role and having appropriate support and practices to manage their well‐being.
9. Ongoing evaluation	Continuous formal assessment and improvement of the YPW programme, ensuring the implementation is effective, equitable and sustainable.
10. Remuneration and policy	State or national youth peer work guidelines that detail YPW's pay and other components of best practice mentioned above.

* YPWs is an abbreviation for ‘youth peer workers.’

A lack of empirical evaluation of these emerging frameworks for understanding peer worker experiences in youth mental health services creates barriers to the safe engagement of YPWs in youth mental health service developments. Identifying the barriers and facilitators to delivering youth peer support from the perspective of YPWs is especially important for optimally integrating YPWs into the mental health system. Moreover, it is timely to use YPW perspectives of their real‐life experiences to refine existing frameworks—a critical step forward for embedding them within mental health services on a larger scale. In our study, we have defined YPWs as peer workers who work specifically in youth mental health services. The aim of our qualitative study was to investigate YPW's experiences of integration into youth mental health services to identify key priorities in implementing YPWs within youth mental health services.

## 1 Methods

### 1.1 Study Design

A qualitative study design was used whereby semi‐structured interviews were conducted with YPWs and general peer workers (who occasionally work with young people) to understand their perspective of how YPWs are being embedded into youth mental health services, including barriers and enablers to the implementation. Themes were analysed using best‐fit framework synthesis based on Australian guidelines on integrating YPWs into mental health services. Author R.S. conducted a rapid narrative synthesis of the Orygen youth peer workforce toolkit [33, 34] and the NSW Mental Health Commission guide for peer workers to identify 10 key components of best practice on the implementation of peer workers (see Table 1). Standards for Reporting Qualitative Research are reported in Supporting Information File 1.

### 1.2 Recruitment

Peer workers within a locally networked group of hospital‐based, community and primary mental health services located in the metropolitan region of Southeastern Sydney, Australia, who worked with young people aged 12–25 years were purposively sampled and invited to participate in the current study. The study was advertised via the organisation's email distribution list. Peer workers who expressed interest by replying to the email were provided with a study information sheet and consent form and given the contact details of the research team so they could discuss any questions. Peer workers were eligible for the study if they worked within a mental health service, even those not working directly with young people. Peer workers were excluded from the study if they were working outside the geographic area of the study and/or worked exclusively with people over the age of 25. We also excluded peer workers from local NGOs as we were interested in embedded models of peer work within the health system. Peer workers were not reimbursed, as interviews occurred during working hours and were seen as part of their contribution to their role.

### 1.3 Theoretical Framework

#### 1.3.1 Study Procedures

Interviews were semi‐structured, 30–60 min long and aimed at understanding the experience of integrating with clinical teams (e.g., ‘how do you find communication with clinical staff?’) and doing peer work with young people (e.g., ‘how did your expectations compare with your experience?’). The question guide (Appendix 1) was developed iteratively through a literature review by the first author, who is a peer worker (R.S.), with input from the wider authorship team, including clinicians and researchers. Author R.S. had a good relationship with the peer workforce, but was supervised by the clinical study leads to consistently manage potential blind spots, conduct reflexivity by exploring subjective interpretations of data and discuss emergent findings. The interviews were conducted by four authors (R.S., M.H., O.A.Y. and S.L.) with divergent backgrounds, described in Box 1. Interviews were recorded via Microsoft Teams and took place either online or in‐person, within meeting rooms at mental health services from which participants were recruited.

Box 1 **Characteristics of the interviewers.**

R.S. is a lived experience project officer with a background in international relations, arts and teaching. She had been employed as a YPW in multiple of the services recruited from for over 2 years and worked with YPWs interviewed. This meant that she possessed an in‐depth, unique understanding of the experience of integration—working to disentangle this from that of the interviewees through reflexive dialogue with M.H.

S.L. is a youth mental health research assistant with a background in psychology who had been embedded within the services for one and a half years. She worked alongside the YPWs interviewed by participating in clinical review meetings and built a sense of rapport with the workforce. Therefore, she has a preliminary understanding of the experiences of YPWs within youth mental health services.

M.H. is a research fellow with a background in teaching, health systems and qualitative research. He had collaborated with services through ongoing consultations on an overarching research project improving the integration of youth mental health services. However, working at the university, he was able to offer an outsider perspective in conversations with the other interviewers.

O.A.Y. is a clinical research assistant with a background in psychology who had been based within the services for 2 years. He worked together with the YPWs interviewed, such as participating in clinical review meetings. This enabled a sense of connection and probing of specific practices but meant that he imbued explanations of cultural dynamics with his own experience, discussing this with the research team throughout.

Study ethics was approved by the SESLHD Human Research Ethics Committee (reference numbers 2022/ETH02721 and 2022/ETH01509). Participants were offered the opportunity to review transcribed digital recordings before they were anonymised, stored in password‐protected folders and coded by the interviewers using a deductive thematic approach [36]. After familiarisation with the dataset, a coding framework was developed based on the components of best practice for implementing a youth peer workforce (Figure 1). Coding using the framework was compared and discussed between R.S., S.L. and M.H. Barriers and facilitators related to each of the 10 best practice components were developed inductively based on a process of recoding the initially coded data.

## 2 Results

### 2.1 Participants

As seen in Table 2, 26 peer workers provided consent to participate in the current study, out of a total of 42 peer workers employed within the health district. Of these participants, 12 peer workers participated in qualitative interviews. Among the participants interviewed, three were from inpatient care, eight from community services and one from other services. Nine of the participants worked in youth‐specific services, while three worked in adult settings. Eight of the participants identified as female, and four identified as male. The ages of participants were not collected and reported in this study to protect confidentiality.

**Table 2:** Demographics of interviewed participants.

Variable	Category	Frequency (*f*)	Percentage (%)
Gender	Male	4	33
	Female	8	67
Service type	Inpatient care	8	67
	Community services	3	25
	Other	1	8
Youth‐specific service	Yes	9	75
	No	3	25

### 2.2 Best‐Fit Framework Analysis of Barriers and Facilitators of Integrating Peer Workers

Table 3 summarises the results discussed below and includes direct quotes from peer workers.

**Table 3:** Barriers and facilitators for youth peer work implementation with key quotes, mapped to best practice components.

Component	Barriers [−] and facilitators [+]	Quotes
1. Role clarity	Generic position descriptions during recruitment [−]	‘If we're putting people into specific areas, they need to have specific roles… mine was just like a generic peer work job description, like it would be for anywhere’. ‘There weren't position descriptions that applied to youth…a lot of the position descriptions talk to inpatients, and the principles may be the same, but the technical component to the roles are very different’.
	Lack of clear day‐to‐day activities, with tasks assigned ad hoc [−]	‘Like I've seen peer workers really struggle when they don't really know what they're meant to do. And then they kind of just get directed by clinical staff who don't know what peer workers are either’.
3. Training	Delayed access to formal peer work training, leading to self‐training [−]	‘It was a lot of personal navigation and personal discovery and a lot of trying to connect with other peer workers’.
	Lack of youth‐specific training [−]	‘There's a gap in upskilling and orientating people to youth peer work, because it's different to adult, [current training] only focuses on adult’.
5. Supervision	Delayed access and lack of peer‐specific supervision [−]	‘If you're going to have a team of peer workers, you need to have a team leader who is a peer worker, who can properly be the first port of call for any peer work‐related questions’.
	Consistent supervision for co‐learning [+]	‘I was doing group supervision and [it was] incredible. That has recently ended so aside from that, I go to a cafe with a couple of other workers that we see once a month’.
7. Professional development	Lack of career progression opportunities [−]	‘I feel like I've had so many opportunities, but when I actually think about growth and moving up in a role, I can't really think of anything.’
	Impacts of limited opportunity for development, such as low motivation, staff retention or lacking senior leadership [−]	‘The reason that I'm leaving is that there's no career progression. I've been advocating actively for the last two years for the creation of a senior peer worker role [unsuccessfully]’.
9. Agency and co‐design	Opportunities to co‐design YPW* roles [+]	‘What's the purpose of having peer workers in this centre? And what roles do we want them to carry out? That should be designed with peer workers’.
	Lack of YPW involvement in the co‐design of youth mental health services [+]	‘I don't feel like the decisions of the centre are made collectively—it's made in private, and then they tell us that it's happening’.
11. Non‐peer education and relationships	Lack of understanding of YPW among non‐peer staff [−]	‘They [non‐peer staff] have no proper induction to what peer work is—no seminar, no education, resources, nothing’. ‘I think some people don't really understand what we do. I know one staff member thinks peer work is eroding the quality of mental health support.’
	Operating in a traditional medical model, where psychology and medicine are valued more highly than YPW [−]	‘I am treated as less than and not taken seriously. I raised on several occasions someone's case manager, that I was worried that they were at risk of hurting themselves. And that was brushed off’.
	Challenging the misunderstanding of YPW [+]	‘I think we have to, as much as possible, try and dismantle the hierarchy that exists within these teams’. ‘This is a work in process. It also depends on who the peer worker is and who the clinician is and what background they have with peer workers. Although it is getting better every day, there is still stigma in certain situations’.
14. Communication	Open communication and feedback with trust and respect [+]	‘I do get really good feedback from clinicians. I do have clinicians asking for my input, or talking about how valuable lived experience discussion is’. ‘I do ask a lot of questions to clinicians to understand the process that they're going through, to have insight around what they might need’.
15. Well‐being	Poor workplace well‐being, linked to discrimination, work stress and lack of proper guidance [−]	‘They can tell that I'm new and younger, so they kind of dismissed me because of that, but there [is] probably some element of stigma or discrimination towards the peer workers, that we're not clinicians, like, how are we supposed to do a proper risk assessment’.
	Supportive management with flexible work practices [+]	‘I like how if I need a sick day, or I need a day off. There's not a lot of questions’.
17. Ongoing evaluation	Lack of programme evaluation effectively measuring YPW [−]	‘I haven't I haven't ever been part of or heard of any sort of evaluation’. ‘I mean, it's really hard to separate and measure outcomes directly from the peer workforce because the young person here is not just receiving treatment from the peer workforce—they're engaging with a psychologist or a GP, or any other clinician. So, it's hard to detach, how much is peer work versus how much is other stuff in a person's life’.
18. Remuneration and policy	Lack of policy underpinning the YPW role [−]	‘I wish there was more of a uniform kind of guidance and things that we could draw on whilst we're finding our feet in the role, because it is such a different way of working’.
	Low remuneration and work hours [−]	‘You know, we do a lot of work and we're paid much less’. ‘We're not getting the coverage that we would like to get. But if they extended our hours, they would get consistency. And they would get variety. And they would get what they deserve, and their patients deserve’.

* YPW is an abbreviation for ‘youth peer work’.

#### 2.2.1 Role Clarity

Peer workers reported two barriers related to role clarity (or lack thereof): *Recruitment*, peer workers indicated that during the hiring process, they were given generic position descriptions that were not youth‐specific. Another major barrier was *lack of clarity of tasks*, whereby peer workers did not have clear day‐to‐day activities. The tasks were given to them in an ad hoc manner, either by clinicians or determined by peer workers themselves. Peer workers commented this affected their confidence in delivering care to consumers and thus had a flow‐on effect on the care of a young person.

#### 2.2.2 Training

Peer workers reported a barrier to integration related to *delayed access to training*. Peer workers reported they did not receive formal training before commencing their work with consumers, except for informal induction by other peer workers. Hence, they often had to self‐discover or copy other peer workers when providing care to consumers. Another barrier was *lack of youth‐specific training* as peer workers noted the formal training they eventually accessed was the Certificate IV in Mental Health Peer Work obtained through Vocational Education and Training [37] and local Recovery College courses [38], which are free workshops provided by the local health district. Neither of these courses is youth‐specific, which peer workers commented they feel is essential to their role.

#### 2.2.3 Supervision

Peer workers identified one major barrier to integration related to *delay in accessing supervision*. All respondents indicated they had received some form of supervision; however, it often took months or years to commence, and they had to advocate for it. Supervision was also often short‐term, given high staff turnover. Further, there were multiple reports of a lack of peer‐specific supervision, where supervisors were not YPWs.

Conversely, peer workers identified *consistent supervision* as a facilitator of integration. Many peer workers reported accessing the same group supervision sessions and spoke highly of the opportunities it provided for co‐learning and reflection. Peer workers stated they were able to discuss unique tensions, especially in the youth specialisation of peer work.

#### 2.2.4 Professional Development

A major barrier to integration was *lack of career progression opportunities*. Peer workers explained the presence of senior peer workers was rare and usually informal, with peer workers only assuming more senior responsibilities over time. There were opportunities to work as a peer worker on the same level but no opportunities for peer workers to extend themselves professionally. A related barrier was identified in relation to the *impact of limited opportunity*. Impacts included lack of motivation for work, low staff retention and a lack of leadership for the current peer workers.

#### 2.2.5 Agency and Co‐Design

The peer workers reported that *co‐design opportunities* would be a facilitator of integration. The benefits of co‐design were particularly noted for the development of peer work position descriptions and functionality in the multidisciplinary team. The peer workers advocated for more lived experience co‐design in youth mental health services in general; however, many seemed to be unsure if the lack of lived experience input had to do with the exclusion of peers or top‐down service design.

#### 2.2.6 Non‐Peer Education and Relationships

Peer workers reported that a barrier to integration was a *lack of understanding among non‐peer staff* about peer workers. Peer workers reported that non‐peer staff needed clarification about how to collaborate and work with peer workers, leading to issues in care coordination, referrals and not seeing peer work as having “equal value” to clinical care. Peer workers also reported being outnumbered in services *operating in a traditional care model* where clinical psychology and medicine are valued more highly than peer work. In the interviews, relationships with non‐peer staff were largely viewed positively by peer workers; however, some struggled to voice their opinions in their organisations and provide good care to consumers.

To address these issues, peer workers indicated that challenging the misunderstanding of peer workers was critical to successful integration. Peer workers referred to the resilience needed to continue to challenge historical structures. Peer workers also reported that, over time, tensions eased, either through building clinical understanding or bonding with non‐peers as a team.

#### 2.2.7 Communication

Peer workers indicated that *open communication* was a facilitator of peer‐worker integration. Some peer workers referred to positive experiences in progressive workplaces where there were open channels of communication with non‐peers, high levels of respect and trust and supportive management. Peer workers reported that some non‐peer staff saw the value of peer work in the youth mental health system. In addition, some peer workers felt open to ask non‐peer staff questions and have open discussions about how they can collaborate with each other.

#### 2.2.8 Well‐Being

Peer workers identified that poor well‐being in the workplace was a barrier to the integration of peer workers. Peer workers mentioned their poor well‐being was linked to discrimination in the workplace, work stress and lack of proper guidance and supervision. Peer workers also mentioned they faced triggering situations and fluctuations in their mental health journey during their day‐to‐day work.

Conversely, peer workers indicated that *supportive management* was important to the management of poor well‐being in the workplace. Some peer workers conveyed they had managers and teams who were responsive and understanding of their mental health and flexible work practices existed for them.

#### 2.2.9 Ongoing Evaluation

*Lack of programme evaluation* was identified as a barrier to the integration of peer workers since none of the participants indicated they were involved in any ongoing evaluation. Furthermore, it was noted that it is been difficult to figure out an effective way of measuring the impact the peer worker has on the service. With a lack of evaluation, peer workers felt as though their services were ‘tokenistic’ with services hoping to attract more funding, and there was no intention to grow the youth peer workforce.

#### 2.2.10 Remuneration and Policy

*Lack of policy* was also identified as another barrier to integration. Peer workers indicated there was no comprehensive YPW or peer worker policy at the state or national level that underpinned their role. Furthermore, peer workers mentioned their pay grade was at the Health Education Officers Graduate/Non‐Graduate under state health awards, which is unrelated to peer rights and responsibilities. Due to a lack of policy, peer workers' roles were also funded temporarily, via various sources, such as philanthropy funds and pilot programme grants. *Low remuneration* was also identified as another major barrier in this domain, with evidence that peer workers' pay grade was not related to their profession. In particular, peer workers viewed their low relative pay to other clinical staff in MDTs as not reflective of their workload. In addition, many peer workers were employed on a part‐time salary, and hence, they expressed a desire for more hours, not only for increased remuneration but also to fulfil the job requirements, including providing continuity of care to consumers.

## 3 Discussion

To develop a unified framework for best practice for youth peer work, the current study investigated the key barriers and facilitators of integrating YPWs into youth mental health services from the experiences of general and youth‐specific peer workers. Best‐fit framework synthesis identified that lack of role clarity, training, limited understanding from non‐peer staff, policy and remuneration were key barriers to the integration of YPWs into youth clinical settings. We identified that successful integration of youth peer work was associated with strong communication networks between peers, non‐peer staff relationships and consistent supervision. The results are discussed in relation to promoting best practice for supporting YPWs within youth mental health services.

The key barriers identified in this study suggest that YPWs require more support and role clarity at each stage of the employment pathway, from recruitment and training to engagement with daily tasks. Key barriers allude to the potential for improving role clarity by having youth‐specific and role‐specific position descriptions while applying for positions. It is recommended that YPWs are provided with a clear framework around the expectations and responsibilities of their role on a day‐to‐day basis, distinct from clinical and other non‐peer roles [7, 11, 12, 34]. Once YPWs begin working, immediate access to training can alleviate concerns associated with peer workers feeling unable to fulfil job tasks. Previous research identified a lack of regular training can culminate in low self‐efficacy, task readiness and competence [21, 39]. To address this, training should be multifaceted by including theoretical training, practical training, peer supervision and ongoing coaching [7, 21, 39, 40].

Barriers to job satisfaction and a sense of being part of the team related to a lack of role understanding among non‐peer staff, lack of centralised policy and low remuneration for peer staff. These findings are consistent with previous research highlighting tensions between non‐peer and peer staff [8, 32]. Non‐peer staff are often hesitant to push workload and accountability onto YPWs due to concerns regarding privacy, professional boundaries and confidentiality of consumers. Because of this, YPWs often felt ignored and under‐appreciated by other staff members [8]. Past research suggests this discord can be attributed to the failure of the whole service to change their delivery of clinical practice to incorporate person‐centred principles and acknowledge the need for lived experience within the clinical journey [7, 11]. There is currently a lack of policy guidelines, including the absence of a specialised salary grading for YPWs due to the poor quality of research evidence currently available [9]. It is important for the field to continuously and systematically evaluate the implementation of youth peer support. This will enable improvement, increase understanding of the value of YPWs and influence policy [34].

In relation to facilitators to effective integration of YPWs into mental health services, the current results revealed the importance of YPWs developing strong relationships with non‐peers. As past research suggests, support and acceptance from colleagues can influence the cultural shift from the medical model to one that acknowledges lived experience over time [41]. Past implementation programmes have strengthened non‐peer relationships by providing staff with continual training about working with and supervising YPWs, addressing concerns and identifying the main differences and similarities peer work has with clinical roles [7, 42]. Another solution is establishing a common language style between peers and non‐peers, such as adopting clinical terms that everyone can understand during meetings [41]. Additionally, a community of peer workers can promote and enhance the well‐being of YPWs. Peers can understand, relate and provide support to each other whilst navigating the uncertainties of their role [11, 12, 41]. Supervision can build these support systems by creating a safe space for peers to bond, co‐learn and reflect on their experiences [8]. Successful supervision can also instil confidence in YPWs by providing ongoing training, on‐the‐job coaching and continuous feedback about their work [41].

The results of the current study provide preliminary support for the utility of a unified framework of best practice for integrating YPWs into mental health services. The current results suggest that 10 key components putatively represent the mechanisms for effective and safe integration of YPWs into clinical settings. However, the current results further suggested that the key components of best practice should be contextualised within pathways of employment for peer workers, allowing for a stage‐by‐stage implementation of the framework's recommendations. For example, cross‐mapping the best practice components with the stages of employment will enable targeted implementation of recommendations, for example, using clearly defined position descriptions during recruitment. Whilst the Orygen youth peer workforce toolkit [33, 34] and NSW Mental Health Commission adult peer workforce toolkit [35] allow for stage‐by‐stage implementation, they have not systematically evaluated and considered multiple YPW perspectives. Furthermore, the key components to integration are not clearly identified in these guidelines. Instead, a series of practical tips are presented which may be confusing to some stakeholders without understanding the basic elements of best practice and its definitions. Mapping the components of best practice is especially important for evaluation purposes. To date, there is no standardised way of evaluating the best practice implementation of YPWs into mental health systems that have been tested for utility, as identified in the review by de Beer et al. While Mutschler et al. [43] synthesise recommendations using the Consolidated Framework for Implementation Research in their systematic review of implementing peer support in mental health services, these are not tailored to YPW and have not been evaluated for appropriateness as in the current study. Additionally, as seen in the NSW Mental Health Commission toolkit [35], other current evaluation guides and tools are derived from broader health and consumer involvement research. Our framework is specifically designed for YPW implementation evaluation in mental health services and, therefore, has the specificity to evaluate the integration more accurately.

The current results against the background of the unified framework also have wider implications for health service policy developments. Policies should consider the best practice components and include mechanisms for formal ongoing evaluations of the overall peer workforce and service‐specific implementation processes. Ongoing evaluation of peer work implementation would strengthen and improve all stakeholders' understanding of the benefits and challenges of the peer support programme for the service's peers and managers. Without a policy across the services, recruitment of YPWs will be conducted without uniformity in their pay, conditions, training and rights. A policy should include guidance on supportive, successful and adaptive youth peer work implementation. A national guideline should encapsulate role clarity, communication, training, supervision, well‐being, culture and leadership [21, 39]. In addition, a national policy could address the lack of recognition and inconsistency of payment and awards peer workers receive in youth mental health services [39].

There are several limitations in the current study for future research to address. Our sample size was small and limited to the recruitment of peer workers employed in one health service district. This limits the generalisability of the results. We propose including the use of quantitative methodology in mixed‐methods designs, such as in the hybrid participatory‐realist evaluation conducted by Halsall et al. [44]. Using larger samples may complement our research through further qualitative exploration of implementation processes and underlying theory, while outcome patterns (e.g., number of peers who are engaged in supervision) are examined through quantitative data to further identify the key change mechanisms for effective and safe integration of YPWs into mental health services. The research should include the perspectives of non‐peer staff as well, which may help to identify how to improve YPWs integration into the wider health ecosystem. Studying different perspectives of professional and end‐user groups will also help reduce bias in results enabling a more accurate and comprehensive understanding of how we can improve integration of YPWs into services. Finally, our brief synthesis of existing frameworks to derive our preliminary best practice framework was limited to the Australian context. This was based on it being representative of the integration of YPWs in primary mental health care settings. A specific programme of primary mental health care for youth populations is a service innovation in Australia and a few other countries (e.g., Ireland and Canada), which means our inferences are limited to these contexts. Future research should seek to replicate the findings in other ambulatory care settings.

## 4 Conclusion

In conclusion, our paper is the first to advocate for using the 10 components of best practice identified in this study to guide and evaluate the implementation of a youth peer workforce to ensure the success and sustainability of this system initiative. These systematic changes in improving the integration of YPWs into the mental health care ecosystem are integral to ensuring the personal lived experience of recovery‐oriented and trauma‐informed approaches is embedded within service delivery, thus helping young people to make sense of the system.

## Author Contributions

**Rose Singh:** conceptualisation, investigation, writing – original draft, methodology, formal analysis, project administration, resources, visualisation, writing – review and editing. **Sarah Leung:** writing – original draft, writing – review and editing, methodology, project administration, investigation, formal analysis, conceptualisation, visualisation, resources. **Vilas Sawrikar:** supervision, writing – review and editing, funding acquisition, writing – original draft, project administration, visualisation. **Catherine McHugh:** writing – review and editing, supervision. **Nan Hu:** formal analysis, supervision. **Oliver Ardill‐Young:** investigation, writing – review and editing, formal analysis, project administration, methodology. **Raghu Lingam:** supervision, funding acquisition, writing – review and editing. **Valsamma Eapen:** writing – review and editing, supervision. **Michael Hodgins:** supervision, funding acquisition, conceptualisation, investigation, methodology, formal analysis, writing – original draft, writing – review and editing. **Jackie Curtis:** supervision, funding acquisition, writing – review and editing.

## Ethics Statement

Study ethics was approved by the SESLHD Human Research Ethics Committee (reference numbers 2022/ETH02721 and 2022/ETH01509).

## Conflicts of Interest

The authors declare no conflicts of interest.

## Supporting information

Supporting information.

## Data Availability

The data that support the findings of this study are available on request from the corresponding author. The data are not publicly available due to privacy or ethical restrictions.

## Appendix 1 Semi‐Structured Interview Questions for Peer Workers

1. What were your expectations about peer work before you began?
  a. Why did you choose to do peer work?
  b. Did you feel confident about doing peer work?
  c. How did your expectations compare to your experience?
2. Do you feel supported in your workplace doing peer work?
  a. Has peer work affected your mental health and well‐being?
  b. Do you feel like your mental health and well‐being are considered in your workplace?
  c. Are there training and development opportunities?
  d. Is there a policy or guidance related to peer work?
  e. Has the peer worker programme been evaluated?
3. How have you found the integration of peer work within your workplace?
4. How do clinical (non‐peer work) staff consider peer work?
  a. How do you find communication with clinical staff?
  b. Are your insights and opinions considered?
  c. Do you feel like you have the capacity to change service practices?
